# Workplace violence experience among nurses at a selected university teaching hospital in Rwanda

**DOI:** 10.11604/pamj.2022.41.64.30865

**Published:** 2022-01-24

**Authors:** Valens Musengamana, Oluyinka Adejumo, Gilbert Banamwana, Marie Josée Mukagendaneza, Thimothée Shahidi Twahirwa, Emmanuel Munyaneza, Jean Damascene Kabakambira

**Affiliations:** 1 School of Nursing and Midwifery, College of Medicine and Health Sciences, University of Rwanda, Kigali, Rwanda; 2 University Teaching Hospital of Kigali, Kigali, Rwanda,; 3 School of Medicine and Pharmacy, College of Medicine and Health Sciences, University of Rwanda, Kigali, Rwanda

**Keywords:** Workplace, violence, nurses, Rwanda

## Abstract

**Introduction:**

workplace violence is a global problem in the health sector, especially in the hospitals affecting healthcare works´ job satisfaction and performance. Workplace violence (WPV) is present in different forms associated with various factors and the nurses are the most affected. This study aimed to explore WPV among nurses working at a selected University Teaching Hospital in Rwanda.

**Methods:**

the research approach used was the quantitative descriptive cross-sectional design. The stratified random sampling was used to recruit 195 participants among 379 nurses. The data were collected using a structured, validated, and self-administered questionnaire that was adapted from the International Labor Organization, International Council of nurses, the World Health Organization, and Public Services International. Descriptive statistics were used for analyzing frequencies and percentages. The Chi-square test was used for evaluating the association between variables.

**Results:**

the findings revealed that (58.5%, n=114) of nurses have experienced some types of WPV in the twelve months preceding the study, among them (44.6%, n=108) of nurses were verbally abused. The nurses providing emergency care, the nurses working in the emergency department, and nurses working with vulnerable patients like HIV/AIDS patients were associated with WPV Chi-square (1, n=195), P=<0.001). The psychological problems have been found to be the first consequences of WPV.

**Conclusion:**

based on the study findings, it was concluded that the hospital management needs to be aware of WPV, develop and implement appropriate policies and strategies. These will strengthen nurses´ concentration towards them and will result in service delivery improvement.

## Introduction

Workplace violence (WPV) in the healthcare system is a worldwide work concern [1]. Workplace is any healthcare facility, whatever the size, location (urban or rural) and the type of service(s) provided, counting major referral hospitals of large cities, regional and district hospitals, healthcare centers, clinics, community health posts, rehabilitation centers, long-term care facilities, general practitioners´ offices, other independent healthcare professionals. In the case of services executed outside the healthcare facility, such as ambulance services or home care, any place where such services are performed will be considered a workplace [2]. In this study, workplace is University Teaching Hospital of Kigali. The World Health Organization (WHO) defined WPV as “the intentional use of power, threatened or actual, against another person or a group, in work-related circumstances, that either results in or has a high degree of likelihood of resulting in injury, death, psychological harm, mal-development or deprivation [3]. The consequences of WPV include physical and psychological negative effects In many studies the consequences of WPV were injuries [4,5] reduction of work performance [6,7], the attainment of institutions´ production [8], leaving the job [9-11] psychological distresses [12], poor work performance [13], demoralization, fearful when working [14], emotional trouble and lack of awareness in a nursing career [15,16].The phenomenon affects all types of healthcare workers [17]. In 2013, the Bureau of Labor Statistics (BLS) in the United States of America (USA) testified that each year WPV attacks about 25,000 individuals of whom most were in healthcare settings and welfare service and its consequences are high compared to the other sector of employees [18]. Different studies were conducted on this subject. In the United State 0f America, two studies revealed that WPV affected 76% and 41% nurses respectively [19]. In Italy, 67% of nurses were reported to have been assaulted (physical and sexual harassment) [12]. In Cyprus, Cypriot Emergency Department, 76% of nurses and doctors were exposed to varied forms of WPV, of which 16% related to verbal abuse, followed by 15% of sexual harassment and 9% of physical violence [20].

In Israeli tertiary hospitals, 58% of staff members have experienced some of WPV [21]. In Palestine hospitals among health care workers in emergency departments, a study revealed a high frequency of WPV (76%) among them physical violence was 36% and 71% of non-physical violence [1]. In China, 13% of nurses and general practitioners suffered from physical violence of which 57% led to injuries while 45% took 2 or 3 days of sick leave [4]. In Jordan, 91% of nurses at the emergency department reported to have experienced WPV [22]. In Saudi Arabia, 36% of nurses reported WPV experience among healthcare workers [6]. In Egypt, 60% of healthcare workers of the Ismailia Hospital´s emergency department reported WPV. Among them 58% were subject to verbal violence while 16% faced physical violence [23]. In Southern Ethiopia, WPV among nurses in public health facilities was 30%. Among them; 90% were verbally abused while 18% were physically violated and 13% of were sexually harassed [17]. In the Gambia, WPV among nurses was 62% [24]. A study conducted in Ghanaian nurses showed that sexual harassment was 12% and verbal abuse was 53% [25]. In Rwanda, the last published study on WPV experience among healthcare workers was in 2011. The findings showed that 39% of participants faced WPV. Of those, 27% were verbally abused, 4% faced physical violence, and 16% experienced psychological violence, while 9% experienced sexually harassment or assault [26]. Most of studies highlighted factors associated with WPV including overcrowding of patients and nurses´ workload, nursing staff shortage, the patients suffering from dementia, or Alzheimer´s illness [22]. Concurrently, overcrowding of patients in need of emergency care [12], working at the emergency department, long waiting time [27], lack of prevention measures, unmet patients and family needs were also reported to expose workers to WPV [1]. Being a young healthcare worker was the most associated with violence as they are still developing nursing skills with least experience in conflict management [10]. Ultimately, being a female was more associated with WPV owing to gender discrimination [17].

Some interventions to address the WPV include training, restricted access, overcrowding reduction, exit strategies [9], having laws, occupational health/safety legislation, and reporting incidents to the relevant authority [28]. Improve nurse-patients communication, conflict management and encouraging respect for health care workers via the mass media were found to decrease the WPV [29]. Development of legal standards, policies and intervention/prevention plans was also adopted [30]. Development of guidelines and toolkit, education program (online training) and reinforce security measures [31]. Job design: adequate staffing, reducing access to cash, avoiding lone working, procedures for raising an alarm or seeking assistance, ensuring patients are kept informed, to limit frustration and misunderstandings, informing patients of waiting times, use a protocol to for summoning assistance [32]. Most strategies to avoid WPV emphasis on education and training program [32,33]. The existing study carried out in Rwandan district hospitals was exploring the WPV in the context of gender discrimination but did not delve into WPV among nurses working in referral hospitals [26]. Therefore, considering the paucity of data related to WPV in Rwandan healthcare settings, this study aimed to explore WPV experience among nurses at a selected University Teaching Hospital in Rwanda and may contribute to the existing improvement evidence-based strategies for its management.

**Objectives:** to assess the types of Workplace Violence, to identify the factors associated with WPV and to explore the consequences of WPV among nurses at a selected University Teaching Hospital. What are the types of WPV experience, the factors associated with WPV and the consequences of WPV among nurses at a selected University Teaching Hospital?

## Methods

**Study design:** the study used a quantitative descriptive cross-sectional design to explore WPV experience among 379 nurses at the University Teaching Hospital of Kigali, Rwanda. The inclusion criteria were; Nurses registered in the nursing council, Nurses interacting with the patients and clients during their work, having more than one year of working experience at a selected University Teaching Hospital in Rwanda and willing to contribute in the study and being on duty throughout the data collection time. The exclusion criteria were; nurses who have not been working at a selected University teaching hospital in Rwanda during past 12 months preceding data collection period because the study reflects the events happening in this period, the participants utilized for the pilot study and the nurses who declined to consent for this study. The exclusion criteria were used to avoid any source of bias. For that reasons, 27 nurses were excluded in this study among them 20 nurses participated in the pilot study and 7 nurses not interacting with the patients and clients (working in Central Sterilization and Supply Department and the Director of Nursing). The population of this study is 406 minus 27 which make an N=379.

**Sampling procedures:** a stratified systematic random sampling was used to obtain 195 participants, recruited in the study in February 2019. Yamane´s formula was used to calculate the sample size. Study participants were divided into 8 departments (strata) including surgical, accident and emergency, internal medicine, critical care (Intensive care unit (ICU) and high dependency units (HDU), pediatrics (including neonatology), outpatient department treatment (OPD), maternity and theater. After that, the proportion was calculated based on the number of nurses in each department divided (N=379) multiplied by 100. The sample in each department was obtained by using obtained sample size of 195 multiplied by the proportion. To get the participants of this study the researcher was used simple random sampling technique, where by an alphabetical list was used to sort out in each department or service, then after one interval was used to get study participants. Data was collected from March 11^th^, 2019 to March 31^st^, 2019 by recalling the WPV experience happened in the twelve months before the study, it was a long period to avoid missing data.

**Operational definitions:** the questionnaire had questions spanning the three types of WPV namely physical violence, psychological violence (verbal abuse and bullying/mobbing) and sexual harassment. Physical violence is defined as the use of physical force against another person or group that results in physical, sexual or psychological harm. It includes among others, beating, kicking, slapping, stabbing, shooting, pushing, biting and pinching. Verbal abuse is a type of psychological/mental abuse that involves the use of oral language, gestured language, and written language directed to a victim. Bullying/mobbing is a repeated and over time offensive behavior through vindictive, cruel or malicious attempts to humiliate or undermine an individual or groups of employee. Sexual harassment is any unwanted, unreciprocated and unwelcome behavior of a sexual nature that is offensive to the person involved, and causes that person to feel threatened, humiliated or embarrassed. Participants who answered “Yes” to at least one question about the above WPV related characteristics were considered as having experienced WPV and were included in the analysis.

**Ethical considerations:** the self-administered questionnaire used was adapted from the instrument elaborated by the International Labor Organization (ILO), International Council of Nurses (ICN), World Health Organization (WHO) and the Public Services International (PSI) [34]. The permission to use the instrument was granted from the ILO Library by email correspondence and was adapted to remain with the questions measuring objectives of the study. The reliability was 0.834 Cronbach´s alpha. To ensure validity of the tool, adoption was used by remaining with the questions measuring each objective, contextualization was used by corresponding each research question to the conceptual framework in order to avoid missing data.

**Statistical analysis:** the statistical methods in this study were selected to describe the features of respondents and exposure to all types of WPV and to find out the consequences of WPV. To identify the factors related with WPV, inferential statistic such us Chi-square test were used to assess relationship between dependent variable (nurse) and independents variables (age groups, sex, working experience, types of patients, sex of patients, working in some specific specialties, work with vulnerable patients, working alone) and WPV. The categorical data was used. Data were analyzed using Statistical Package for the Social Sciences (SPSS) version 21, IBM. Descriptive statistics were used for analyzing frequencies and percentages. A statistical significance was taken at the level of P<0.05. The study was exposed to the panel in the June 2019. The details of the data analysis and the results were displayed in tables.

## Results

**Characteristics of the participants:** all participants (195) responded to the questionnaires. The majority of participants were females at 85%. The ages ranged from 25 to 64 years, most participants were in the age group of 30-39 (50%). Most respondents were married (84%). Most respondents had more than 6 years of working experience (85%) (Table 1).

**Table 1:** demographic data for the study participants

Variables	Categories	Frequency	Percent
**Gender**	Female	165	84.6
	Male	30	15.4
**Age**	25-29 years	12	6.2
	30-34 years	46	23.6
	35-39 years	52	26.7
	40-44 years	47	24.1
	45-49 years	26	13.3
	50-54 years	6	3.1
	55-59 years	4	2.1
	60 -64 years	2	1.0
**Marital status**	Single	25	12.8
	Married	163	83.6
	Living with partner	1	1.5
	Separated or divorced	3	1.5
	Widow or Widower	3	1.5
**Work experience**	1-5 years	29	14.9
	6-10 years	62	31.8
	11-15 years	40	20.5
	16-20 years	26	13.3
	Over 20 years	38	19.5

The majority of participants were females at 85%; the ages ranged from 25 to 64 years, most participants were in the age group of 30-39 (50%); most participants of respondents were married (84%; most respondents had more than 6 years of working experience (85%)

**Types of Workplace violence in the last 12 months:** about 58.5% of nurses have experienced at least one type of WPV. Among them, 55.4% were verbally abused, 15.4% were bullied/mobbed, 6.7% have experienced physical violence, and 2.1% have experienced sexual harassment (Table 2).

**Table 2:** types of workplace violence experienced in the last 12 months

Variables	Categories	
	Yes	No
Some types of WPV	114(58.5%)	81(41.5%)
Physical violence	13(6.7%)	182(93.3%)
Physical violence without a weapon	12(6.2%)	183(93.8%)
Physical violence with a weapon	1(.5)	194(99.5%)
Verbal abuse	108(55.4%)	87(44.6%)
Bullying/mobbing	30(15.4%)	165(84.6%)
Sexual harassment	4(2.1%)	191(97.9%)

58.5% of nurses have experienced at least one type of WPV; among them, 55.4% were verbally abused, 15.4% were bullied/mobbed, 6.7% have experienced physical violence, and 2.1% have experienced sexual harassment

**Factors associated with workplace violence:** a chi-square for independence showed a significant association between working in specific specialties and WPV, Chi2(1, n=195), P=<0.001, with a high WPV to the nurses working in emergency care specialty (mean=1.50), and HIV/AIDS patients (mean=1.11). Furthermore, the results revealed a high association between WPV at accident and emergency department (mean=1.68) (Table 3).

**Table 3:** factors associated with workplace violence

Variables	Categories	Mean	N	Df	Value	P-Value
Gender	Female	0.79	165	3	0.38	0.287
	Male	0.87	30			
Age	25-34 years	1.49	58	21	27.91	0.143
	35-44 years	1.76	99			
	45-54 years	0.97	32			
	55-64 years	1.25	6			
Marital status	Single	0.80	25	12	3.55	0.997
	Married	0.81	163			
	Living with partner	0.00	1			
	Separated or divorced	1.00	3			
Nursing work experience	1-10 years	1.63	91	12	12.75	0.387
	11-20 years	1.68	66			
	Over 20 years	0.63	38			
The category of patients/ clients	Newborns and children	1.13	43	6	5.26	0.511
	Adults	0.85	152			
The sex of patients	Female	1.03	35	6	7.49	0.278
	Male	0.71	7			
	Male and female	0.75	153			
Specialties	Physical disabled	0.38	42		58.96	<0.001
	Mother/child care	0.79	67			
	Terminally ill	0.73	26			
	HIV/AIDS	1.11	6	18		
	Emergency care	1.50	24			
	Chronic disease care	0.85	20			
	Others	0.80	10			
Departments/Services	OPD	1.00	16	21	78.9	<0.001
	Internal medicine	1.03	29			
	Surgical	0.34	35			
	Maternity	0.96	31			
	Pediatrics	0.63	36			
	Emergency	1.68	19			
	Theatre and critical care	0.88	29			
The number of staff present	None	0.90	10	12	17.77	0.123
	1-10	1.41	159			
	11-15	1.00	16			
	Over 15	0.60	10			

A Chi-square for independence showed a significant association between working in specific specialties and WPV, Chi2(1, n=195), P=<0.001, with a high WPV to the nurses working in emergency care specialty (mean=1.50), and HIV/AIDS patients (mean=1.11); furthermore, the results revealed a high association between WPV at accident and emergency department (mean=1.68)

**Consequences of workplace violence:** the most consequences of WPV experience were psychological problems like avoiding thinking about it or talking about the attack or avoiding having feelings interrelated to it (43.6%). The repeated, worrying memories, thoughts, or images of the attack were at the rate of 35.9%, being super-alert or watchful and on guard (11.8%), feeling like everything you did was an effort (11.3%). Thinking of leaving the job (10.8%), reduction of work performance (11.8%), and nurses felt ashamed or guilty after being violated (9.7%). One (0.5%) nurse has been injured as a result of the violence (Table 4).

**Table 4:** consequences of workplace violence

Variables	Frequency	Percent
Repeated, disturbing memories, thoughts, or images of the attack	70	35.9
Avoiding thinking about or talking about the attack or avoiding having feelings related to it	85	43.6
Being super-alert or watchful and on guard	23	11.8
Feeling like everything you did was an effort	22	11.3
Reduction of work performance	21	10.8
Thinking of leaving the job	27	13.8
Felt ashamed or guilty	19	9.7
Injured as a result of the violence	1	0.5

The most consequences of WPV experience were psychological problems like avoiding thinking about it or talking about the attack or avoiding having feelings interrelated to it (43.6%); the repeated, worrying memories, thoughts, or images of the attack were at the rate of 35.9%, being super-alert or watchful and on guard (11.8%), feeling like everything you did was an effort (11.3%); thinking of leaving the job (10.8%), reduction of work performance (11.8%), and nurses felt ashamed or guilty after being violated (9.7%); one (0.5 %) nurse has been injured as a result of the violence

## Discussion

**Types of workplace violence in the last 12 months:** the study indicated that 58.5% of nurses have experienced at least one type of WPV. However, the frequency is lower compared to the studies conducted in the Gambia (62.1%) [24], China 66% [35] and Malawi (70.54%) [15] but higher compared to the studies conducted in Italy (45%) [12] and Saudi Arabia (36%) [6]. This slight difference may be due to the increasing numbers of nurse-patients´ ratio, initiation of incident reporting management and the implementation of policies and procedures related to security and safety which are being adopted in work place. Concerning the types of WPV, 6.7% of nurses experienced physical violence. This rate is low compared to the studies conducted in some countries; in North West of Ethiopia (60.2%) [8], in Democratic Republic of Congo (53.6%) [36], in the Texas State (USA) (49.8%) [9], in Malawi (22%) [15]. In South Ethiopia, physical violence was for 18%, [17] in the Gambia (17.2%) [24], in China (around 12%) [35], in Lebanon (10%) [10]. In this study, it was found that the presence of security staff at all time, reducing the overcrowding of patients, summoning assistance in case of security threat and initiation of non-violence culture in the country might be contributing to the reduction of physical violence among 93.3% of nurses. The findings of the present study revealed that 44.6% of nurses were verbally abused.

These results are low compared to the studies conducted in different countries, in Malawi (95%) [15], in the South of Ethiopia (90%) [17] in China (65%) [35] in Lebanon (62%) [10], in Gambia (59.8%) [24], in the Democratic Republic of Congo (59.0%) [36] and Ghana (52.7%) [25]. Therefore, being in the accreditation process and applying customer care standards and regular patients´ satisfaction survey might decrease the level of this violence. The results showed that 15.4% of nurses were bullied/ mobbed. These findings are also low compared to the studies conducted in different countries, in Malawi (73%) [15], in the North West of Ethiopia (39.8%) [8]. This discrepancy could be linked with reporting bullying and mobbing on time, working in a team, intensive supervision to resolve the conflict between nurses/patients which might decrease the violence and contribute to the physical violence. These strategies similarly were used in China [37]. Lastly, the findings showed that 2.1% of nurses have experienced sexual harassment, this frequency is very low compared to the studies conducted in the Democratic Republic of Congo (63.3%) [36], in Texas State (USA) (46%), [9] in Malawi (16%) [15], in South of Ethiopia (13%) [17], in Ghana (12.2%) [25], and Gambia (10%) [24]. The vast majority (97.9%) of nurses who were not exposed to sexual harassment might be explained by the strict application of gender-based violence law in the country. It was demonstrated that the evidence-based strategies to manage WPV involve having laws, occupational health, and safety legislation, offering in-service training, education, and reporting them to the relevant authority [28].

**Factors associated with workplace violence:** the study revealed a statistically significant association between working in a specific specialty and WPV, Chi2(1, n=195), P=<0.001, with a high WPV to the nurses working in emergency care specialty (mean=1.50), and HIV/AIDS patients (mean=1.11), an association between working in accident and emergency department (mean=1.68). The results of this study are similar to those found in Italy, where, working in the emergency department was the most associated with WPV [12] and in South Ethiopia [17]. Therefore, working with suffering persons generates WPV since they are likely frustrated and irritated due to their illness and pain. It was found that suffering persons are impatient in the matter of waiting time as long as they are often expecting to receive punctual care [38]. Violence is common amongst healthcare workers in contact with them and it is frequently reflected as an unavoidable part of the occupation [38]. This is the case of the nurses giving emergency care, or working in accident and emergency and for nurses working with patients with HIV/AIDS. However, the factors with an insignificant p-value were marital status, Chi2(1, n=195), P=0.997, the category of patients/clients, Chi2(1, n=195), P=0.511, nursing work experience Chi2(1, n=195), P=0.387, sex of patients, gender, Chi2 (1, n=195), P=0.289, the sex of patients, Chi2 (1, n=195), P=0.278, nurses´ ages, Chi2 (1, n=195), P=0.143, and the number of staff present in the workplace Chi2 (1, n=195), P=0.123. The findings are in contrast with the study conducted in Iraq where age, marital status, gender, work experience were associated with WPV [13]. Therefore, in Taiwan age and working experience were not associated with WPV [13]. In China age was not associated with WPV and work experience was associated with WPV [35]. The Palestinian study showed also absence of association between gender and WPV [35]. In the Northwest Ethiopia sex of patients of patients was not associated with WPV, however working in male ward, the number of staff in the same shift were associated with WPV [8].

**Consequences of workplace violence:** the consequences of WPV among nurses highlighted in the study were related to psychological distress among others; avoiding thinking about or talking about the attack or avoiding having feelings related to it 43.6%, repeated, disturbing memories, thoughts, or images of the attack at a rate of 35.9%, being super-alert or watchful and on guard 11.8%, feeling like everything you did was an effort 11.3%, reduction of work performance 11.8% and nurses felt ashamed or guilty after being violated 9.7%. These findings are supported by previous studies conducted in Italy, where psychological distresses were reported to be over 73% [12]. In addition, our findings are supported by studies conducted in Ghana, where complaints of repeated disturbed memories and feelings or images of the abuse were 33.8% [25]. The study conducted in Saudi Arabia indicated that the decreasing in job productivity was (31.1%) [6]. In Palestine psychological distress was 9.3%) while feelings of guilt was 1.3% [1]. Finally, in Lebanon, nurses had high levels of emotional exhaustion and depersonalization [10]. It was found that WPV generates psychological concerns like depressive symptoms [39], and this might correlate with the results of this study. The study discovered that 10.8% of nurses thought of leaving their job. This frequency is low compared with the studies conducted in Lebanon, where 31.7% thought to leave their nursing job [10], also in Palestine [1], in North of Ethiopia the reduction of institutions productions [8]. It is clear-cut evident that the WPV can influence nurses´ intention to leave their job [40], the intension to quit the work might due to psychological concerns.

**Limitation of study:** firstly, the study was steered in one of the University Teaching Hospitals in the entire country and was limited to one category of healthcare workers (nurses). Secondly, the study used a quantitative approach only. We acknowledge that an additional qualitative method would have provided a better understanding. Finally, the study used a retrospective self-reporting approach in data gathering, by recalling events in the last twelve months prior to the study. Such studies in general are susceptible to recall biases. Consequently, these findings may not be generalized to the whole hospital settings in Rwanda but sheds light on the current WPV situation as the phenomenon of workplace violence in the health sector is transversal and similarities in the violence against healthcare professionals worldwide have been demonstrated in previous studies.

## Conclusion

Workplace violence is a significant problem among nurses working at CHUK. Working in the emergency department and working with vulnerable patients such as those living with HIV/AIDS was highly associated with WPV. Psychological distress is likely to be the most consequences of WPV. The development and implementation of policies and strategies at the hospital at both hospital and national level are needed. These strategies could enable healthcare workers to be more productive while on job resulting in service delivery improvement.

### What is known about this topic

- *There was a single study conducted in Rwanda on exploration of WPV. The study was conducted in district hospitals in the context of gender discrimination and was published in 2011;*
- *The study is old and doesn’t provide information about WPV among nurses working in referral hospitals*.

### What this study adds

- *We discovered different types, risk factors and consequences of WPV among nurses working at a selected University Teaching Hospital in Rwanda;*
- *We discovered that 58.5% of nurses have experienced some types of WPV which is an alarming situation;*
- *It may be argued that the research is not robust because we did not manage to make it multicenter due to financial limitations, but the study sets the stage for further studies in the field and will inform policy makers*.
